# Sociodemographic Disparities and Reasons for Delayed Healthcare Among U.S. Cancer Survivors: An All of Us Study

**DOI:** 10.21203/rs.3.rs-7698480/v1

**Published:** 2025-11-18

**Authors:** Ding Quan Ng, Zhiyuan Zheng, Ahmedin Jemal, Alexandre Chan, Farhad Islami

**Affiliations:** University of California, Irvine; American Cancer Society; American Cancer Society; University of California, Irvine; American Cancer Society

## Abstract

**Objective:**

To examine how sociodemographic factors influence healthcare access among cancer survivors.

**Methods:**

From the National Institutes of Health’s All of Us dataset (2018–2022, n = 27,589), we analyzed the relationship between characteristics like age, income, race/ethnicity, and insurance, and reasons for delayed healthcare, including affordability, transportation, and nervousness.

**Results:**

The top reasons for delayed care were affordability issues (12%), nervousness (8%), and transportation barriers (6%). Younger survivors (ages 18–39), those on Medicaid, and individuals earning less than $25,000 annually consistently experienced higher rates of delayed care. Female survivors were more likely to delay care for all reasons except transportation. Work and caregiving-related delays were more common among minoritized racial/ethnic groups, while non-Hispanic White survivors more often delayed care due to nervousness and socioeconomic factors.

**Conclusions:**

Considerable differences in delayed healthcare were observed among cancer survivors by sociodemographic characteristics, with evidence for intersectionality for several observed associations. Findings highlight the need for personalized patient navigation strategies to effectively address the unique social needs of each cancer survivor, ultimately improving healthcare access for all.

## Introduction

Cancer survivors require consistent and timely healthcare engagement across the care continuum^1^. Delayed and inadequate survivorship follow-up care can lead to poorer survival, wellness, and quality of life, adoption of adverse lifestyle behaviors, late detection of recurrences, and suboptimal control of complications, such as cardiomyopathy, fatigue, pain, neuropathy, psychological distress, and neurocognitive impairments^2–6^.

There are many reasons why cancer survivors would forgo or delay medical care. High out-of-pocket costs and medical financial hardships are most frequently investigated as they directly affect patient’s ability to pay for healthcare services, especially for individuals with lower incomes and without health insurance^7–9^. Other barriers include lack of sick leave from workplace, family responsibilities, lack of transportation, and fear of stigma^10–13^. The effects of each barrier to healthcare are multidimensional, as each may interplay with other demographic, social, economic, and cultural factors. As such, tailoring patient navigation strategies to an individual’s social needs is likely to be more effective in addressing healthcare access barriers^14^.

Previous studies have examined disparities in health-related social needs, medical financial hardship, and health outcomes among cancer survivors^15–17^. Few studies, however, have evaluated disparities across a variety of potential reasons for delayed healthcare, as a function of patient-level characteristics and their interactions, with adequate statistical power. Understanding these aspects of access-to-care barriers can help provide tailored care to cancer survivors.

To address this knowledge gap, we aimed to comprehensively evaluate the association of sociodemographic characteristics, individually and collectively, with major reasons for delayed healthcare among cancer survivors.

## Methods

The All of Us Research Program has been administered by the National Institutes of Health (NIH) since May 2018 to facilitate biomedical research in accordance with the Precision Medicine Initiative launched in 2015^18,19^. For this study, we used data from the All of Us Controlled Tier Dataset version 7 (C2022Q4R9). Eligible participants for the current study included those who completed the “Health Care Access & Utilization” survey and were diagnosed with cancer, either self-reported or with ≥ 2 EHR diagnosis records of cancer (ICD-9-CM: 140 to 209, ICD-10-CM: C00 to C96) prior to survey completion. Participants who were diagnosed with only skin cancer were excluded. Data on self-reported biological sex, race/ethnicity, educational attainment, annual household income, employment, health insurance, marital status, and homeownership status were obtained from the “Health Care Access & Utilization” survey^20^. Based on the date of completion of the outcome questionnaire in that survey and self-reported date of birth, participants’ age at the time of survey was calculated. All research data are organized in the Observational Health and Medicines Outcomes Partnership (OMOP) common data model v5.2^21^.

Within the “Health Care Access & Utilization” survey, participants were also asked about delayed healthcare: “*There are many reasons people delay getting medical care. Have you delayed getting care for any of the following reasons in the past 12 months*?” These reasons included “*could not afford co-pay, could not afford deductible, need of out-of-pocket payment, could not get time off work, provided care to an adult, could not get childcare, did not have transportation, and were nervous about seeing a provider*”. For each listed reason for delayed healthcare, the following responses were made available: “Yes”, “No” or “Don’t know”. We combined reasons related to out-of-pocket payment, co-pay, and deductible expenditure into one category as “affordability”.

### Statistical analysis

The distribution of sociodemographic characteristics was presented with overall counts and percentages, stratified by reasons for delayed healthcare. Using logistic regression, associations between evaluated sociodemographic characteristics and reasons for delayed healthcare were examined, overall and after stratification by age group (18–39, 40–64, 65 + years), biological sex (female, male), race/ethnicity (non-Hispanic White [NHW], non-Hispanic Black [NHB], Hispanic/Latinx), and cancer type (four leading causes of cancer deaths^22^: breast [female only], prostate, colorectal, lung). Group differences between strata were evaluated with Z-test for comparing two independent regression coefficients $\left(Z=\frac{b_{1}-b_{2}}{\sqrt{{SEb}_{1}^{2}-{SEb}_{2}^{2}}}\right)$^23,24^. Effect sizes were presented as odds ratios (OR) and 95% confidence intervals (CI) with the largest category serving as the reference group for each categorical characteristic. Statistical significance in the adjusted models with all cancer survivors were determined by a Bonferroni-corrected significance level of 0.000397 *[0.05/ (21 covariates × 6 reasons for delayed healthcare)]*. All other analyses were similarly two-tailed but tested at 5% significance level. We accessed the data and performed all analyses using R (version 4.1.0) in a cloud-based platform operated on Jupyter Notebook.

## Results

Of 413,360 participants in the All of Us Controlled Tier Dataset version 7 through July 1st, 2022, a total of 27,589 cancer survivors were included in this analysis (**eFigure 1**). The majority of cancer survivors were 65 years or older (55.7%), female (61.9%), identified as NHW (81.9%), attained above high school education (88.4%), had private health insurance (57.4%), and were married (59.9%) and homeowners (74.7%) (Table 1).

### Reasons for delayed healthcare

The top reasons for delayed healthcare were affordability problems with out-of-pocket healthcare expenditure (12.4%), nervousness about seeing a provider (8.3%), and lack of transportation means (5.6%) (Table 1). Cancer survivors aged 18–39 years, enrolled in Medicaid, and earning less than $25,000 a year were consistently among the top 3 strata with the highest prevalence of delayed healthcare across the evaluated reasons (Table 2).

In Bonferroni-corrected adjusted analyses of the full cohort, delayed healthcare was associated with age at survey (due to all 6 reasons), biological sex (all reasons except transportation), health insurance (all reasons except nervousness), income (affordability, transportation, and nervousness), employment and homeownership status (affordability, work, and transportation), marital status (childcare and transportation), and race/ethnicity (nervousness). In contrast, delayed healthcare was not associated with education attainment for any reason. Table 3 details these findings, which are summarized in **eTable 1**.

### Age and delayed healthcare

**Figure 1:**
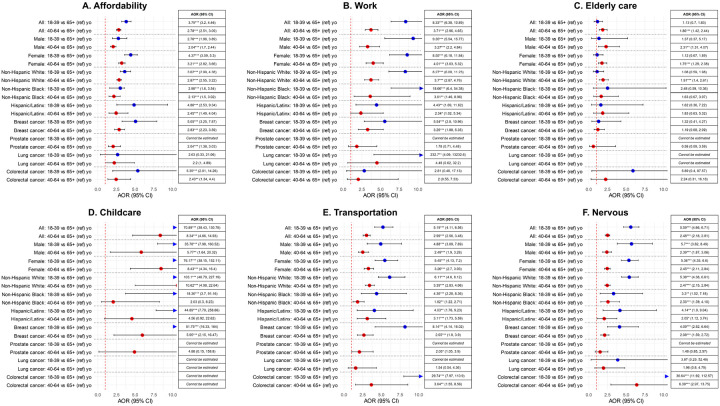
Association between age group (reference group = 65+ years old) and various barriers underlying delayed healthcare across select strata. Abbreviations: AOR, adjusted odds ratio; CI, confidence interval. *P<0.05, **P<0.01, ***P<0.001.

Younger age was consistently associated with higher odds of delayed healthcare for all evaluated reasons (Fig. 1, Table 3, **eTable 1**). The youngest group (i.e., 18–39 years, adolescent and young adult, AYA) had consistently higher odds of delayed healthcare (Fig. 1, **eTables 2–6**). Younger colorectal cancer survivors were more prone to delaying healthcare due to nervousness about seeing a provider (Fig. 1, **eTable 6**).

When stratified by age group, not having health insurance (reference = private insurance) was more strongly associated with delayed healthcare due to affordability and transportation barriers among AYAs than older cancer survivors (**eTable 2**). Additionally, NHB AYAs were more likely to report delayed healthcare due to work-related barriers than their NHW counterparts, but this difference was not observed in older age groups (40–64 and 65 + years old) (**eTable 2**). However, a few associations were stronger in older ages: delayed healthcare was associated with work-related barriers among Hispanic/Latinx (reference = NHW) survivors aged 65 + years, childcare barriers among home renters (reference = owners) aged 40–64 years, transportation barriers among unmarried/separated (reference = married) survivors aged 65 + years, and affordability barriers among survivors with <$25,000 annual household income (reference=$50,000 to <$100,000) in ages 40 + years (**eTable 2**).

### Biological sex and delayed healthcare

**Figure 2:**
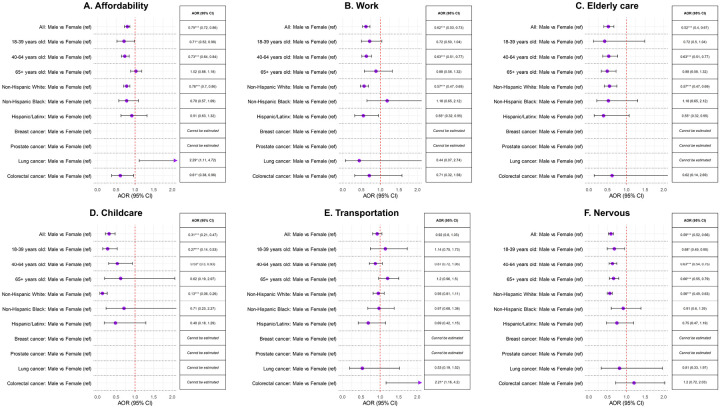
Association between male biological sex (reference group = female) and various barriers underlying delayed healthcare across select strata. Abbreviations: AOR, adjusted odds ratio; CI, confidence interval. *P<0.05, **P<0.01, ***P<0.001.

Across all strata, male cancer survivors had consistently lower adjusted odds of delayed healthcare for all investigated reasons than females or odds were similar, with 2 exceptions (Fig. 2, Table 3, **eTable 1**). Compared to their female counterparts, males with lung cancer had 2.29-times (95% CI = 1.11–4.72) higher odds of reporting affordability-related delayed healthcare and males with colorectal cancer had 2.21-times (95% CI = 1.16–4.20) higher odds of transportation-related delayed healthcare (Fig. 2, **eTables 5–6**).

In analyses stratified by biological sex, female cancer survivors who rented their homes were more likely to delay healthcare due to affordability barriers and nervousness, compared to female homeowners. This difference was not as pronounced within male cancer survivors (**eTable 3**). Additionally, a younger age was a stronger predictor of affordability-related delayed healthcare among females compared to males (**eTable 3**). In contrast, some characteristics were more strongly associated with delayed healthcare among male cancer survivors, including work-related delays among NHB individuals (reference = NHW), childcare-related delays among non-Hispanic Asian and/or Native Hawaiian or Pacific Islander (NHA/NHPI) individuals (reference = NHW) and home renters (reference = owners), and nervousness-related delays among unmarried/separated individuals (reference = married) (**eTable 3**).

### Race/ethnicity and delayed healthcare

**Figure 3:**
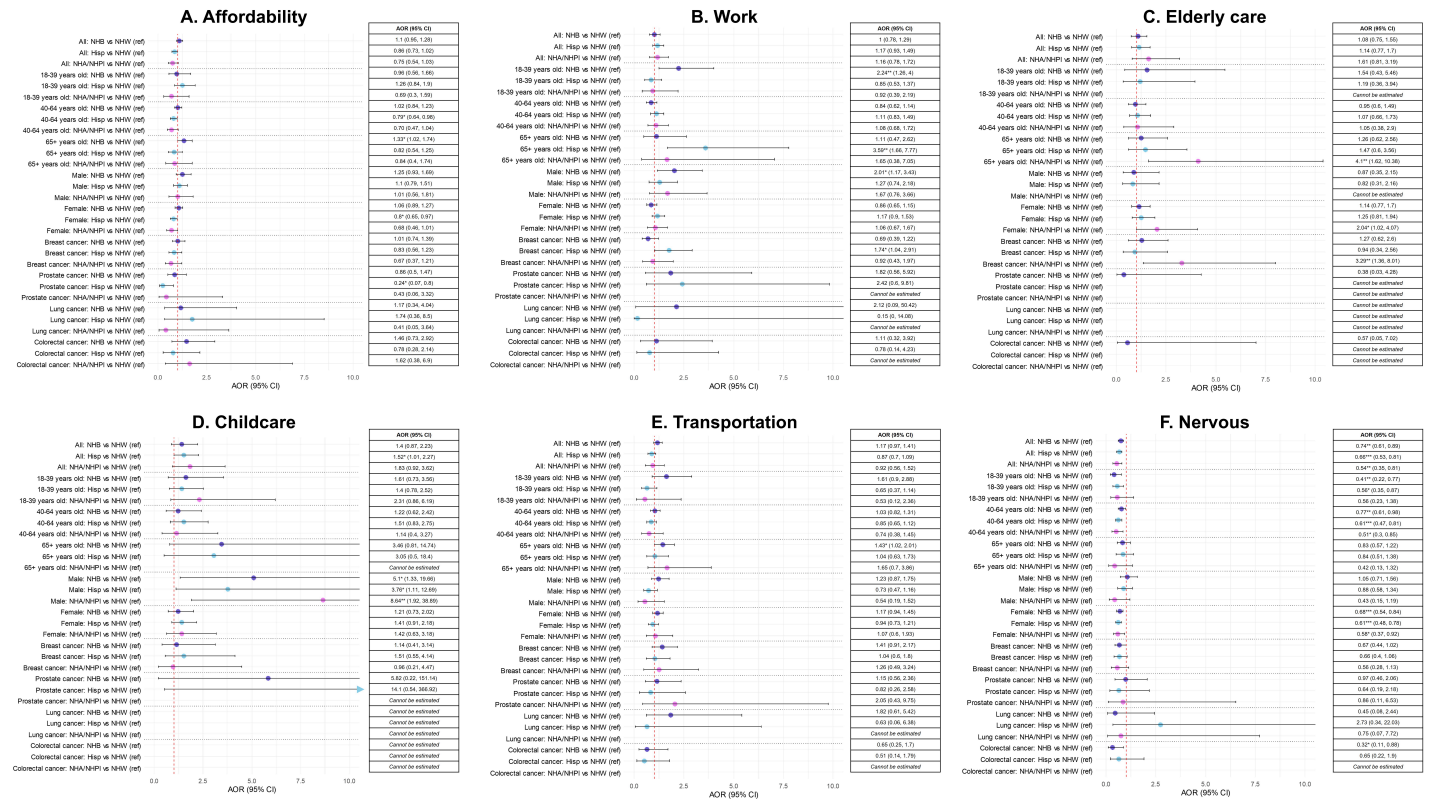
Association between minority racial/ethnic groups (reference group = NHW) and various barriers underlying delayed healthcare across select strata. Abbreviations: AOR, adjusted odds ratio; CI, confidence interval; Hisp, Hispanic/Latinx; NHA, non-Hispanic Asian; NHB, non-Hispanic Black; Native Hawaiian/Pacific Islander, NHPI. *P<0.05, **P<0.01, ***P<0.001.

Compared to NHW, NHB and Hispanic/Latinx individuals had higher odds of delaying healthcare in unadjusted analyses, but these associations were largely attenuated in adjusted models with Bonferroni correction. The only association that persisted in adjusted models was lower odds of delaying healthcare due to nervousness to see a provider among Hispanic/Latinx than NHW cancer survivors (OR = 0.66, 95% CI = 0.53–0.81) (Fig. 3, Table 3, **eTable 1**). This association was consistently observed in ages < 65 years and among females (Fig. 3, **eTables 2 and 3**).

While race/ethnicity was not associated with other delayed care reasons in full cohort analyses, some significant associations were observed in stratified analyses (Fig. 3). For example, compared to their NHW counterparts, NHB AYA and NHB male cancer survivors were more likely to report delayed care due to work (Fig. 3, **eTables 2 and 3**). NHB cancer survivors also had higher odds of delayed care due to affordability and transportation barriers in older age groups compared to NHW survivors (Fig. 3, **eTable 2**). Hispanic cancer survivors were more likely to delay healthcare than NHW survivors due to work-related barriers among older adults and breast cancer survivors (Fig. 3, **eTables 2 and 5**). NHA/NHPI individuals were more likely than NHW to delay healthcare due to elderly care among older adults, females, and breast cancer survivors (Fig. 3, **eTables 2, 3 and 5**).

Compared to females, male cancer survivors were generally less likely to delay healthcare due to work, childcare and nervousness to see a provider, with much lower odds among NHW than NHB (all above 3 reasons) and Hispanic (childcare) cancer survivors (**eTable 4**). In contrast, the association between being uninsured or covered by Medicaid (reference = private insurance), unemployed (reference = employed), or renting (reference = owning a home) and delayed healthcare due to affordability, childcare, transportation, and work-related barriers were significantly greater among NHW than among NHB cancer survivors (**eTable 4**).

### Other sociodemographic characteristics and delayed healthcare

**Figure 4:**
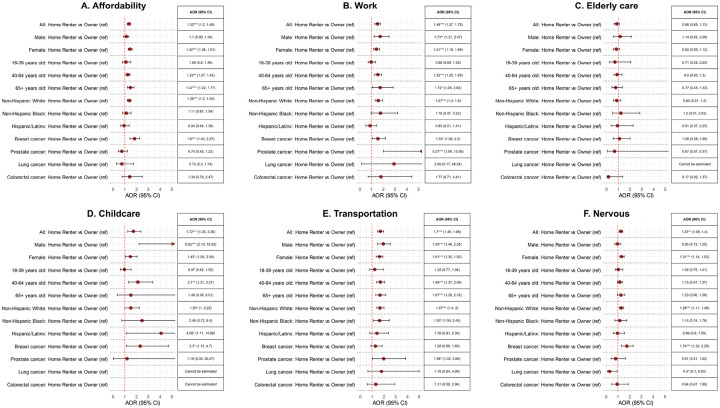
Association between home renters (reference group = homeowners) and various barriers underlying delayed healthcare across select strata. Abbreviations: AOR, adjusted odds ratio; CI, confidence interval. *P<0.05, **P<0.01, ***P<0.001.

Home renters were at higher odds of reporting delayed healthcare due to affordability, work, and transportation barriers than homeowners even after adjustment for education attainment, household income, employment, and health insurance status and Bonferroni correction (Fig. 4, Table 3, **eTable 1**). Findings were generally consistent in analyses stratified by sociodemographic characteristics (Fig. 4, **eTables 2–6**).

Other notable characteristics that were associated with higher odds of delayed healthcare after Bonferroni correction included elderly care, childcare, and transportation barriers among Medicaid enrollees (reference = private insurance); affordability and transportation barriers among individuals with annual household incomes of <$50,000 (reference=$50,000 to <$100,000); transportation barriers among non-workers (reference = employed); and transportation barriers among Medicare/Dual eligibility enrollees and uninsured survivors (reference = private insurance, reason = affordability), and unmarried/separated survivors (reference = married, reason = transportation) (Table 3, **eTable 1**).

## Discussion

In this large, nationwide study of cancer survivors in the United States (U.S.), we found considerable differences in delayed healthcare and its reasons by age group, biological sex, and homeownership status that persisted after adjustments for multiple sociodemographic characteristics. There were variations in the strengths of these associations across combinations of sociodemographic characteristics, indicating intersectionality. This information can provide additional insight into factors contributing to disparities in access to care and opportunities for reducing these disparities, improving survival and survivorship outcomes, and enhancing quality of life across evaluated populations.

As with previous studies^25–27^, we found that younger cancer survivors generally experience more barriers to healthcare access. Especially for AYAs, receiving a cancer diagnosis can be a life-changing event at a key transitional phase of life, as it can interfere with their pursuit of higher education, career progression, financial security, and family planning^28^. Moreover, AYAs are more likely to have limited health insurance coverage and wealth compared to older adults^29,30^, and consequently, are more likely to experience cancer-related financial toxicity than older cancer survivors^31^. Similarly, delayed healthcare due to work-related factors can stem from prioritizing early career development while coping with productivity-reducing effects of cancer, ongoing survivorship care needs, and challenges with meeting expectations amidst fear of discrimination based on medical history^32–34^. The significant gap in healthcare access between privately insured and uninsured AYAs in this study underscored the importance of insurance enrollment as a modifiable factor. Current health systems and policies remain inadequate to facilitate access to care for younger cancer survivors with these unique needs^35–38^. The demand for cancer care in younger adults is likely to grow given the increasing incidence of several cancer types in this population, such as colorectal and endometrial cancer^39,40^. Crucially, young age is a stronger predictor of nervousness before a medical appointment among colorectal cancer compared to other cancers in our study, suggesting significant unmet psychosocial needs in this population. Increasing insurance coverage, including the adoption of Medicaid expansion in all states, can alleviate access-to-care barriers in AYAs^41^. Efforts may also be placed on facilitating paid sick leaves, hybrid education and work arrangements to allow flexibility of time for younger cancer survivors to seek the necessary care they require despite their busy schedules^42–45^. Other interventions that could increase healthcare engagement include setting up childcare facilities within the vicinity of cancer centers and developing specialized clinics to address and accommodate the unique needs of younger cancer patients with small children^46,47^.

In this study, female cancer survivors were more likely than males to experience delays in healthcare for several reasons, including affordability, work responsibilities, elderly care, childcare, and nervousness about seeing a provider. This pattern highlights the unique challenges women face as they balance professional duties and caregiving roles. Other studies have similarly reported higher financial toxicity among female than male cancer survivors^48–50^. Increased nervousness with seeing healthcare providers among females may in part stem from negative experiences with previous healthcare encounters^51–53^. Together, these challenges increase the risk of follow-up loss with healthcare providers. Interventions elaborated earlier to help younger cancer survivors for addressing delayed healthcare may also help with sex-related disparities. Other priority actions may include co-designing health systems that cater to care needs of girls and women, establishing a framework for gender competence in the healthcare workforce, and enhancing avenues for women’s involvement in research and leadership^54^. Nevertheless, male cancer survivors may also experience greater delays in care compared to their female counterparts in certain subpopulations. In this study, males with lung or colorectal cancer were more likely to experience delayed healthcare because of affordability and transportation barriers, respectively, than females. This observation could in part be explained by the lower socioeconomic status among male lung cancer survivors compared to females^55^. More research is required on transportation barriers among colorectal cancer survivors^56–58^.

By race/ethnicity, higher impact of work-associated barriers on NHB AYAs and older Hispanic/Latinx survivors suggest that these groups may experience limited access to flexible work arrangements to navigate cancer treatment and survivorship. Elderly care commitments weighed more heavily on older, female, and breast cancer survivors in the NHA/NHPI population, a finding consistent with existing literature and underscoring the critical need for targeted interventions for cancer survivors who are also caregivers^59–61^. Male cancer survivors from minoritized racial/ethnic groups were more likely than NHW males to delay healthcare due to childcare responsibilities, an often-overlooked intersection of biological sex, race/ethnicity, and caregiving burden in healthcare access. Nevertheless, NHW survivors were more prone to delaying healthcare due to provider-related nervousness than minoritized racial/ethnic groups, underscoring the need for tailored interventions. The impact of lower socioeconomic status (e.g., being uninsured, covered by Medicaid, unemployed, or renting) on healthcare delays was also significantly greater among NHW survivors, revealing another frequently unaddressed interaction of race/ethnicity and socioeconomic status within healthcare access.

Our findings reemphasize the importance of housing security as a key social determinant of health^62^. A previous study reported that newly diagnosed cancer survivors with housing instability concerns were at two-times higher mortality risk after adjusting for other social risk factors^63^. Owning a house provides a stable place to live in and indicates the availability of wealth to liquidate or mortgage in case of emergent expenditure^64^. On the other hand, renters may face financial constraints due to rising rental costs in many parts of the U.S., which may lead to a prioritization of work and immediate financial needs over investing in cancer care^65^. Transitioning between accommodations could also lead to losses to follow-up care among renters^65^. In this study, the impact of not owning a house on delayed healthcare was significantly greater in cancer survivors aged 40 and over, indicating considerable unmet needs among less wealthy individuals in this group. Certain interventions could reduce housing insecurity and its adverse effects on health. At the providers’ level, routine screening for housing insecurity is recommended to ensure timely referrals to community resources to address cancer survivors’ needs. This entails strong connections between cancer centers with community-based safety net organizations^62^. For dealing with housing instability from the repeated changing of accommodations, enabling telemedicine services and interoperability of healthcare institutions could be implemented in environments with limited access to healthcare^66,67^.

A key strength is its large sample size, which enables robust, comprehensive analyses. However, a cross-sectional design is a limitation of this study, as reverse causality may be a concern with any observed associations. In addition, the use of self-report data increases the risk of recall bias that could lead to over- or under-estimation of effect sizes. Racial-ethnic groups other than those included in this study, minoritized sexual-gender groups, and unhoused populations are under-represented in the data that were used in this study. As such, reasons for delayed healthcare in these under-represented populations should be further evaluated. Considering the evidence of intersectionality influencing delayed healthcare among cancer survivors in this study, coupled with limited intersectionality research across the cancer care continuum^68,69^, future studies should consider applying the framework of intersectionality to evaluate and develop interventions at reducing health disparities and inequities in cancer care.

## Conclusion

Delayed healthcare among cancer survivors varies significantly by age, sex, and homeownership, with intersectional patterns shaping these disparities. To promote equitable access, tailored interventions are essential. Expanding insurance coverage, such as nationwide Medicaid expansion, offering financial navigation services, and supporting paid sick leave and flexible work arrangements may help reduce barriers. Future research should evaluate these strategies through an intersectional lens to address the complex challenges faced by diverse survivor populations.

## Supplementary Material

Supplementary Files

This is a list of supplementary files associated with this preprint. Click to download.

- SupplementaryOnlineContent09232025.docx

## Figures and Tables

**Table 1.** Sociodemographic characteristics among cancer survivors in the All of Us Research Program, stratified by reasons for delayed healthcare

Variable	*Overall* *(N=27,589)*	Reasons for delayed healthcare in the past 12 months					
		Affordability^1^ (N = 3500)	Work (N = 1271)	Elderly care (N = 433)	Childcare (N = 324)	Transportation (N = 1540)	Nervous (N = 2282)
Prevalence, %	100%	12.7%	4.6%	1.6%	1.2%	5.6%	8.3%
Age at survey, n (%^3^)							
18–39	*1541 (5.6%)*	439 (28.5%)	316 (20.5%)	32 (2.1%)	165 (10.7%)	227 (14.7%)	396 (25.7%)
40–64	*10,686 (38.7%)*	2025 (19.0%)	835 (7.8%)	254 (2.4%)	NR^4^	872 (8.2%)	1246 (11.7%)
65+	*15,362 (55.7%)*	1036 (6.7%)	120 (0.8%)	147 (1.0%)	≤ 20	441 (2.9%)	640 (4.2%)
Biological sex, n (%^3^)							
Male	*10,196(37.9%)*	905 (8.9%)	231 (2.3%)	86 (0.8%)	36 (0.4%)	415 (4.1%)	504 (4.9%)
Female	*16,651 (61.9%)*	2468 (14.8%)	993 (6.0%)	333 (2.0%)	274 (1.6%)	1065 (6.4%)	1706 (10.2%)
Race/ethnicity, n (%^3^)							
NH White	*21,765 (81.9%)*	2547 (11.7%)	878 (4.0%)	283 (1.3%)	191 (0.9%)	970 (4.5%)	1729 (7.9%)
NH Black	*1851 (7.0%)*	311 (16.8%)	100 (5.4%)	46 (2.5%)	31 (1.7%)	235 (12.7%)	169 (9.1%)
Hispanic/Latinx	*1475 (5.6%)*	253 (17.2%)	121 (8.2%)	43 (2.9%)	41 (2.8%)	137 (9.3%)	145 (9.8%)
NH Asian/NHPI	*461 (1.7%)*	54 (11.7%)	33 (7.2%)	≤ 20	≤ 20	23 (5.0%)	30 (6.5%)
Educational attainment, n (%^3^)							
High school diploma or less	*3125 (11.6%)*	471 (15.1%)	143 (4.6%)	79 (2.5%)	55 (1.8%)	386 (12.4%)	343 (11.0%)
Some college	*6624 (24.7%)*	1082 (16.3%)	362 (5.5%)	136 (2.1%)	94 (1.4%)	527 (8.0%)	652 (9.8%)
Bachelor	*7552 (28.1%)*	959 (12.7%)	378 (5.0%)	101 (1.3%)	86 (1.1%)	327 (4.3%)	614 (8.1%)
Master or more	*9558 (35.6%)*	874 (9.1%)	351 (3.7%)	102 (1.1%)	78 (0.8%)	241 (2.5%)	610 (6.4%)
Annual household income, n (%^3^)							
Less than $25,000	*3335 (12.1%)*	666 (20.0%)	148 (4.4%)	127 (3.8%)	75 (2.2%)	675 (20.2%)	504 (15.1%)
$25,000 – $49,999	*4079 (14.8%)*	749 (18.4%)	240 (5.9%)	88 (2.2%)	58 (1.4%)	276 (6.8%)	358 (8.8%)
$50,000 – $99,999	*7247 (26.3%)*	901 (12.4%)	338 (4.7%)	92 (1.3%)	75 (1.0%)	209 (2.9%)	526 (7.3%)
$100,000 – $199,999	*6266 (22.7%)*	579 (9.2%)	286 (4.6%)	46 (0.7%)	50 (0.8%)	104 (1.7%)	428 (6.8%)
$200,000 and above	*2903 (10.5%)*	167 (5.8%)	117 (4.0%)	≤ 20	27 (0.9%)	41 (1.4%)	170 (5.9%)
Missing	*3759 (13.6%)*	438 (11.7%)	142 (3.8%)	NR^4^	39 (1.0%)	235 (6.3%)	296 (7.9%)
Employment status, n (%^3^)							
Employed	*10,359 (38.6%)*	1701 (16.4%)	1064 (10.3%)	139 (1.3%)	153 (1.5%)	368 (3.6%)	1010 (9.7%)
Not working	*16,479 (61.4%)*	1685 (10.2%)	174 (1.1%)	277 (1.7%)	157 (1.0%)	1109 (6.7%)	1201 (7.3%)
Health insurance, n (%^3^)							
Private	*15,415 (57.4%)*	2009 (13.0%)	958 (6.2%)	174 (1.1%)	166 (1.1%)	452 (2.9%)	1263 (8.2%)
Medicare/Dual eligibility	*8208 (30.5%)*	780 (9.5%)	67 (0.8%)	119 (1.4%)	26 (0.3%)	499 (6.1%)	470 (5.7%)
Medicaid	*1840 (6.9%)*	305 (16.6%)	124 (6.7%)	90 (4.9%)	95 (5.2%)	396 (21.5%)	340 (18.5%)
Uninsured (including IHS only, single service plans)	*388 (1.4%)*	140 (36.1%)	31 (8.0%)	13 (3.4%)	≤ 20	55 (14.2%)	55 (14.2%)
Marital status, n (%^3^)							
Married	*16078 (59.9%)*	1729 (10.8%)	619 (3.8%)	187 (1.2%)	185 (1.2%)	456 (2.8%)	1101 (6.8%)
Divorced/Separated/Widowed	*6719 (25.0%)*	957 (14.2%)	292 (4.3%)	135 (2.0%)	60 (0.9%)	598 (8.9%)	575 (8.6%)
Non-married	*2981 (11.1%)*	512 (17.2%)	228 (7.6%)	73 (2.4%)	40 (1.3%)	336 (11.3%)	393 (13.2%)
Living with partner	*1062 (4.0%)*	181 (17.0%)	91 (8.6%)	21 (2.0%)	28 (2.6%)	82 (7.7%)	138 (13.0%)
Homeownership, n (%^3^)							
Owner	*19934 (74.7%)*	2050 (10.2%)	698 (3.5%)	237 (1.2%)	134 (0.7%)	570 (2.9%)	1314 (6.6%)
Renter	*466 (20.5%)*	1064 (19.5%)	430 (7.9%)	116 (2.1%)	139 (2.5%)	701 (12.8%)	691 (12.6%)
Cancer types^2^, n (%^3^)							
Breast (female only)	*5566 (20.2%)*	685 (12.3%)	253 (4.5%)	94 (1.7%)	62 (1.1%)	226 (4.1%)	468 (8.4%)
Prostate	*2822 (10.2%)*	211 (7.5%)	30 (1.1%)	≤ 20	≤ 20	72 (2.6%)	102 (3.6%)
Colorectal	*943 (3.4%)*	130 (13.8%)	38 (4.0%)	≤ 20	≤ 20	66 (7.0%)	93 (9.9%)
Lung	*535 (1.9%)*	49 (9.2%)	≤ 20	≤ 20	≤ 20	30 (5.6%)	36 (6.7%)

Abbreviations: NH, non-Hispanic; NR, not reported; NHPI, Native Hawaiian, Pacific Islander.

1 Delayed healthcare due to affordability reasons includes: could not afford co-pay, could not afford deductible, and need of out-of-pocket payment.

2 Exclude multiple cancers.

3 Row percentages are presented for all columns other than the “Overall” column, where they present column percentages.

4 NR cells are masked even though they have > 20 counts to prevent the back-calculation of cells with ≤ 20 counts in compliance with the All of Us Data and Statistics Dissemination Policy.

**Table 2:** Top three sociodemographic characteristics with highest prevalence of delayed healthcare for each reason among cancer survivors in the All of Us Research Program

Reasons for delayed healthcare in the past 12 months	Overall prevalence, %	Prevalence Rank 1	Prevalence Rank 2	Prevalence Rank 3
		Subgroup, %		
Affordability^1^	12.7%	Age: 18–39 (28.5%)	Income: <$25k (20.0%)	Homeownership: Renter (19.5%)
Work	4.6%	Age: 18–39 (20.5%)	Employment: Employed (10.3%)	Marital status: Living with partner (8.6%)
Elderly care	1.6%	Insurance: Medicaid (4.9%)	Income: <$25k (3.8%)	Insurance: Uninsured (3.4%)
Childcare	1.2%	Age: 18–39 (10.7%)	Insurance: Medicaid (5.2%)	Race/ethnicity: Hispanic (2.8%)
Transportation	5.6%	Insurance: Medicaid (21.5%)	Income: <$25k (20.2%)	Age: 18–39 (14.7%)
Nervous	8.3%	Age: 18–39 (25.7%)	Insurance: Medicaid (18.5%)	Income: <$25k (15.1%)

1 Delayed healthcare due to affordability reasons includes: could not afford co-pay, could not afford deductible, and need of out-of-pocket payment.

**Table 3:** Odds ratios (95% confidence intervals) for the association between sociodemographic factors and delayed healthcare among cancer survivors in the All of Program

	Affordability^1^		Work		Elderly care		Childcare		Transportation		Nerve
	Unadj.	Adj.	Unadj.	Adj.	Unadj.	Adj.	Unadj.	Adj.	Unadj.	Adj.	Unadj
**Age at survey**											
18–39	4.58*** (4.03–5.21)	**3.79** *** ^§^ **(3.20–4.46)**	29.78*** (23.93–37.05)	**8.33** *** ^§^ **(6.38–10.89)**	1.83** (1.24–2.69)	1.13 (0.70–1.83)	90.94*** (55.02–150.30)	**70.89** *** ^§^ **(38.43–130.78)**	5.78*** (4.88–6.85)	**5.19** *** ^§^ **(4.11–6.56)**	7.54** (6.56–8.67)
40–64	2.95*** (2.72–3.20)	**2.78** *** ^§^ **(2.51–3.09)**	10.16*** (8.37–12.32)	**3.71** *** ^§^ **(2.96–4.65)**	2.28*** (1.86–2.80)	**1.86** *** ^§^ **(1.42–2.44)**	11.01*** (6.65–18.22)	**8.34** *** ^§^ **(4.66–14.93)**	3.00*** (2.67–3.38)	**2.9** *** ^§^ **(2.56–3.48)**	2.94 (2.66–3.24)
65+	1.00	1.00	1.00	1.00	1.00	1.00	1.00	1.00	1.00	1.00	1.00
**Biological sex**											
Female	1.00	1.00	1.00	1.00	1.00	1.00	1.00	1.00	1.00	1.00	1.00
Male	0.57*** (0.53–0.62)	**0.79** *** ^§^ **(0.72–0.86)**	0.37*** (0.32–0.42)	**0.62** *** ^§^ **(0.53–0.73)**	0.43*** (0.34–0.54)	**0.52** *** ^§^ **(0.40–0.67)**	0.22*** (0.15–0.31)	**0.31** *** ^§^ **(0.21–0.47)**	0.62*** (0.55–0.69)	0.92 (0.80–1.05)	0.45** (0.41–0.50)
**Race/ethnicity**											
NH-White	1.00	1.00	1.00	1.00	1.00	1.00	1.00	1.00	1.00	1.00	1.00
NH-Black	1.59*** (1.39–1.81)	1.10 (0.95–1.28)	1.42** (1.14–1.75)	1.00 (0.78–1.29)	1.99*** (1.45–2.74)	1.08 (0.75–1.55)	1.99*** (1.36–2.92)	1.40 (0.87–2.23)	3.18*** (2.73–3.70)	1.17 (0.97–1.41)	1.22* (1.03–1.44)
Hispanic	1.53*** (1.33–1.77)	0.86 (0.73–1.02)	2.14*** (1.75–2.60)	1.17 (0.93–1.49)	2.25*** (1.62–3.12)	1.14 (0.77–1.70)	3.23*** (2.29–4.55)	1.52* (1.01–2.27)	2.25*** (1.86–2.71)	0.87 (0.70–1.09)	1.28** (1.07–1.53)
NH-Asian/NHPI	0.98 (0.74–1.32)	0.75 (0.54–1.03)	1.81** (1.26–2.60)	1.16 (0.78–1.72)	1.50 (0.76–2.93)	1.61 (0.81–3.19)	3.00*** (1.66–5.43)	1.83 (0.92–3.62)	1.13 (0.74–1.73)	0.92 (0.56–1.52)	0.80 (0.55–1.16)
**Education attainment**											
High school diploma or less	1.80*** (1.59–2.03)	0.99 (0.85–1.14)	1.30* (1.06–1.58)	1.11 (0.88–1.41)	2.44*** (1.81–3.28)	0.97 (0.68–1.38)	2 21*** (1.56–3.13)	0.67 (0.42–1.05)	5.58*** (4.72–6.59)	1.42*** (1.15–1.74)	1.89** (1.64–2.17)
Some college	1.96*** (1.78–2.16)	1.19** (1.07–1.34)	1.54*** (1.33–1.79)	1.25* (1.05–1.49)	1.95*** (1.50–2.52)	0.96 (0.72–1.29)	1.75*** (1.30–2.37)	0.84 (0.54–1.31)	3.37*** (2.88–3.93)	1.34** (1.12–1.61)	1.63** (1.45–1.83)
Bachelor	1.43*** (1.29–1.57)	1.09 (0.98–1.22)	1.38*** (1.19–1.60)	1.08 (0.92–1.28)	1.23 (0.93–1.63)	0.89 (0.67–1.20)	1.38* (1.01–1.87)	0.89 (0.64–1.25)	1.75*** (1.48–2.07)	1.16 (0.96–1.40)	1.31** (1.16–1.47)
Master or more	1.00	1.00	1.00	1.00	1.00	1.00	1.00	1.00	1.00	1.00	1.00
**Annual household income**											
Less than $25,000	1.78*** (1.59–1.99)	**1.62** *** ^§^ **(1.39–1.88)**	0.97 (0.80–1.19)	1.25 (0.96–1.64)	3.09*** (2.35–4.06)	1.78** (1.24–2.55)	2.22*** (1.60–3.06)	0.84 (0.54–1.31)	8.73*** (7.42–10.26)	**2.78** *** ^§^ **(2.26–3.42)**	2.36** (2.08–2.69)
$25,000 - $49,999	1.63*** (1.47–1.82)	**1.62** *** ^§^ **(1.44–1.83)**	1.32** (1.12–1.57)	1.30* (1.06–1.59)	1.75*** (1.30–2.35)	1.52* (1.10–2.08)	1.41 (1.00–1.99)	0.92 (0.62–1.38)	2.47*** (2.05–2.97)	**1.59** *** ^§^ **(1.30–1.94)**	1.26** (1.09–1.45)
$50,000 - $99,999	1.00	1.00	1.00	1.00	1.00	1.00	1.00	1.00	1.00	1.00	1.00
$100,000 - $199,999	0.69*** (0.62–0.77)	**0.62** *** ^§^ **(0.55–0.70)**	0.95 (0.81–1.12)	0.83* (0.70–1.00)	0.56** (0.39–0.79)	0.56** (0.38–0.81)	0.75 (0.52–1.07)	0.68 (0.46–1.01)	0.56*** (0.44–0.72)	0.68** (0.53–0.87)	0.91 (0.80–1.04)
$200,000 and above	0.41*** (0.35–0.49)	**0.35** *** ^§^ **(0.29–0.43)**	0.84 (0.68–1.04)	0.70** (0.55–0.89)	0.31*** (0.17–0.58)	0.32*** (0.17–0.60)	0.88 (0.56–1.37)	0.96 (0.59–1.57)	0.48*** (0.34–0.67)	0.66* (0.46–0.94)	0.77** (0.64–0.92)
**Employment status**											
Employed	1.00	1.00	1.00	1.00	1.00	1.00	1.00	1.00	1.00	1.00	1.00
Not working	0.62*** (0.57–0.66)	**0.76** *** ^§^ **(0.69–0.84)**	0.10*** (0.08–0.11)	**0.16** *** ^§^ **(0.13–0.20)**	1.34** (1.09–1.65)	1.15 (0.90–1.46)	0.68*** (0.55–0.85)	1.50** (1.13–1.99)	1.98*** (1.76–2.23)	**1.86** *** ^§^ **(1.60–2.16)**	0.75** (0.69–0.82)
**Health insurance**											
Private	1.00	1.00	1.00	1.00	1.00	1.00	1.00	1.00	1.00	1.00	1.00
Medicare/Dual eligibility	0.74*** (0.67–0.80)	1.00 (0.89–1.12)	0.13*** (0.10–0.17)	**0.53** *** ^§^ **(0.39–0.72)**	1.36** (1.08–1.72)	1.38* (1.03–1.85)	0.31*** (0.20–0.47)	1.03 (0.61–1.73)	2.16*** (1.89–2.46)	**1.56** *** ^§^ **(1.32–1.85)**	0.70** (0.63–0.78)
Medicaid	1.31*** (1.14–1.49)	**0.54** *** ^§^ **(0.46–0.64)**	1.10 (0.91–1.34)	0.73* (0.56–0.94)	4.46*** (3.44–5.79)	**1.94** *** ^§^ **(1.37–2.74)**	4.98*** (3.85–6.44)	**2.38** *** ^§^ **(1.61–3.52)**	9.26*** (8.01–10.72)	**1.49** *** ^§^ **(1.23–1.81)**	2.60** (2.28–2.97)
Uninsured (including IHS only, single service plans)	3.99*** (3.19–4.98)	**2.07** *** ^§^ **(1.59–2.69)**	1.32 (0.91–1.92)	0.82 (0.52–1.28)	3.07*** (1.73–5.45)	1.80 (0.96–3.37)	3.46*** (1.98–6.04)	2.70** (1.40–5.21)	5.62*** (4.16–7.60)	1.59* (1.12–2.26)	1.91** (1.42–2.55)
**Marital status**											
Married	1.00	1.00	1.00	1.00	1.00	1.00	1.00	1.00	1.00	1.00	1.00
Divorced/Separated/Widowed	1.42*** (1.31–1.55)	0.95 (0.86–1.06)	1.17* (1.01–1.35)	1.12 (0.94–1.33)	1.79*** (1.43–2.23)	0.88 (0.68–1.13)	0.79 (0.59–1.07)	0.57** (0.40–0.82)	3.40*** (3.00–3.85)	1.51***^§^ (1.29–1.76)	1.32** (1.19–1.47)
Non-married	1.67*** (1.50–1.86)	0.81* (0.71–0.93)	2.06*** (1.76–2.41)	0.97 (0.80–1.18)	2.06*** (1.57–2.72)	1.03 (0.75–1.42)	1.13 (0.80–1.60)	**0.26** *** ^§^ **(0.17–0.39)**	4.39*** (3.79–5.08)	1.30* (1.08–1.56)	2.07** (1.83–2.34)
Living with partner	1.64*** (1.38–1.94)	0.98 (0.81–1.18)	2.32*** (1.84–2.92)	1.13 (0.87–1.47)	1.64* (1.04–2.59)	0.94 (0.58–1.54)	2 22*** (1.48–3.32)	0.55* (0.34–0.89)	2.88*** (2.26–3.68)	1.09 (0.82–1.44)	2.02** (1.67–2.45)
**Homeownership**											
Owner	1.00	1.00	1.00	1.00	1.00	1.00	1.00	1.00	1.00	1.00	1.00
Renter	211*** (1.95–2.29)	**1.33** *** ^§^ **(1.20–1.48)**	2.38*** (2.10–2.69)	**1.49** *** ^§^ **(1.27–1.75)**	1.78*** (1.42–2.23)	0.86 (0.65–1.13)	3.82*** (3.01–4.86)	1.72*** (1.25–2.36)	5.04*** (4.50–5.66)	**170** *** ^§^ **(1.46–1.98)**	2.08* (1.89–2.30)

Abbreviations: Adj., adjusted; IHS, Indian health service; NH, non-Hispanic; NHPI, Native Hawaiian, Pacific Islander; Prev, prevalence; Unadj., unadjusted.

1 Delayed healthcare due to affordability reasons includes need of out-of-pocket payment, too high deductibles and cannot afford co-pay.

* P < 0.05,

** P < 0.01,

*** P < 0.001.

§ P < 0.000397 (Bonferroni-corrected significance level) for adjusted analyses.

## Data Availability

The All of Us Research Hub employs a tiered data access model with three levels (Public Tier, Registered Tier, and Controlled Tier). The Controlled Tier includes data from electronic health records (EHRs), wearables, surveys, and physical measurements collected at participant enrollment, as well as genomic data from whole-genome sequencing and genotyping arrays. Unlike the Registered Tier, restricted demographic data fields from EHRs and surveys, and unshifted event dates are made available in the Controlled Tier. Currently, the Controlled Tier data are accessible to researchers affiliated with academic institutions, non-profit organizations, and health care institutions (both non-profit and for-profit), with plans to extend access to additional groups, including industry-affiliated researchers. To access the Controlled Tier, researchers must complete the All of Us Researcher Workbench access process, which includes identity verification and specific training on human participant research (https://www.researchallofus.org/register/). Researchers may create a workspace at any time to conduct studies, provided they comply with Data Use Policies and self-declare their research purpose. This information is publicly available on the All of Us Research Projects Directory at https://allofus.nih.gov/protecting-data-and-privacy/research-projects-all-us-data.
